# History of Cholera in the Vicinity of Victoria, Knox Co. Ill.

**Published:** 1851-03

**Authors:** J. W. Spalding


### ARTICLE X.

*History of Cholera in the vicinity of Victoria, Knox Co, Ill.* By J. W. Spading, M. D.

In compliance with my promise, I proceed to give a history of the cholera as it occurred in this vicinity in the summer and fall of 1849. It was observed and commonly remarked by the profession, that there was an uncommon tendency to irritation of the bowels, accompanied with diarrhœa during the spring and summer. In fact almost every case of disease had the above complication.

In a colony of Swedes fifteen miles from here, the disease broke out in August, and the mortality was uncommonly great. Out of a population of about 250, there was 115 fatal cases in the course of two or three weeks. They were treated by one of the steam and pepper gentry, and he had excellent luck in running his patients into the ground, for not one recovered, and he pronounced the malady incurable. The above colony had frequent communication with the river towns where the disease was prevalent. In addition to this, a company of Norwegians arrived, by the Lakes, and Illinois canal, when the disease spread over them as with the besom of destruction.

On Sept 12th, I was called to see a Swede woman, (who had just arrived by the above route, and was stopping with a Swedish family two miles from this place,) and found her with a train of symptoms that satisfied me it was Cholera. Her disease was controlled, and in two days she was convalescent. On Sunday morning, Sept. 16th, I was called to see a young Swede man at the same place, and found him in the stage of collapse. He was taken that morning, and when I arrived he was cold, with profuse cold perspiration, no pulsation at the wrists, his eyes sunken to the bottom of the sockets, the skin of the face drawn tight over the bones, the hands very much corrugated ; the vomiting had ceased, but he was passing rice water from the bowels. The treatment was external warmth with frictions, cal. opii. camp., with stimulants. He continued to sink and died in the evening. Another man living at the same place was attacked on Wednesday, Sept. 19th, at 4 A. M., and died at 10 A. M. On Friday, Sept. 21st, the wife of the last was attacked, and died the same day. Two other Swedes at the same place were attacked but recovered.

The disease subsided until about the 10th of October, when on the arrival of some Swedes from the east, it broke out again, and a number of cases proved fatal. Being absent I cannot give you particulars. Only one American, however, died. I am not much of a contagionist, but must confess the above cases favor the idea that it originated in that way.

There has been but one case of cholera at the Swedish colony this season. A man returned from St. Louis and died with it without propagating the disease.

